# Piezo1-mediated autophagy promotes immune-inflammatory responses in ankylosing spondylitis

**DOI:** 10.1038/s41419-025-08230-7

**Published:** 2026-01-08

**Authors:** Hui Zhao, Xueying Yu, Minxin Jiang, Ziqi Li, Yanyu Zhao, Yuxin Ren, Lanlan Fang, Mengmeng Wang, Xiaofeng Lu, Yubo Ma, Guosheng Wang, Qiang Zhou, Yanfeng Zou, Guoqi Cai, Faming Pan

**Affiliations:** 1 Department of Epidemiology and Biostatistics, School of Public Health, Anhui Medical University, Hefei, Anhui China; 2 The Inflammation and Immune Mediated Diseases Laboratory of Anhui Province, Anhui Medical University, 81 Meishan Road, Hefei, Anhui 230032 China; 3 The First Affiliated Hospital of Anhui Medical University, Anhui Public Health Clinical Center, Hefei, Anhui China; 4 Department of Clinical Laboratory, the Second Hospital of Anhui Medical University, Hefei, Anhui China

**Keywords:** Autoimmune diseases, Chronic inflammation

## Abstract

This study aimed to investigate the role of Piezo1 in the immune-inflammatory response during the pathogenesis of ankylosing spondylitis (AS) and its underlying mechanisms. RT-qPCR was used to evaluate the expression levels of Piezo1 and autophagy-related genes in the peripheral blood of AS patients. Correlation analyses were conducted to evaluate associations between Piezo1 expression and clinical characteristics of AS patients. Immunohistochemistry (IHC) and Western blotting were performed to determine the expression of Piezo1 and autophagy-related proteins in the synovium of AS patients. In vitro, the effects of Piezo1 and autophagy on primary monocyte-derived macrophages and fibroblast-like synoviocytes (FLS) from AS patients were investigated using Yoda1 and 3-MA interventions. Modulation of the immune-inflammatory response via the Piezo1-autophagy axis was examined through RT-qPCR, Western blotting, and ELISA. Finally, the role of Piezo1 in immune regulation was further assessed in proteoglycan-induced arthritis (PGIA) model using GsMTx4, a Piezo1 inhibitor. Piezo1 expression was markedly elevated in AS patients compared to healthy controls and showed a positive correlation with disease activity, duration, and autophagy levels. Mechanistically, increased Piezo1 expression induced M1 polarization of monocyte-macrophages, leading to increased autophagy and the upregulation of inflammatory factors. Additionally, Piezo1 enhanced autophagy and IL-6 activation in FLS. In the PGIA model, GsMTx4 inhibited autophagy hyperactivation, significantly reduced the immune-inflammatory response, and exerted a protective effect on spinal bone tissue. Immunofluorescence further confirmed that these effects might be associated with reduced autophagy and inflammatory cytokine expression in macrophages and FLS. These findings highlight the role of Piezo1 in AS pathogenesis, suggesting that Piezo1 may contribute to the immune-inflammatory response in AS through autophagy regulation.

## Introduction

Ankylosing spondylitis (AS) is a chronic autoimmune disorder marked by sacroiliac arthritis, primarily affecting the axial skeleton [1]. Clinically, it presents with peripheral arthritis, inflammatory low back pain, and osteophytes, which can lead to ossification and spinal deformity [2]. AS predominantly affects young males. Although non-steroidal anti-inflammatory drugs (NSAIDs), glucocorticoids, and biologics (e.g., TNF-α inhibitors) have significantly improved clinical symptoms [3], spinal fractures and imaging progression remain common in patients with longer disease duration [4]. Thus, further investigation of the predisposing factors and underlying mechanisms of AS is essential.

Mechanotransduction is an evolutionarily conserved signal transduction mechanism that enables eukaryotes to sense and respond to physical signals from both internal and external environments, converting them into biochemical signals [5, 6]. In mammals, it plays a role in touch perception, nociception, immune regulation, bone homeostasis, and autophagy [7–9]. Recently, the role of mechanotransduction in the immune system has garnered significant attention. Mechanotransduction is believed to be crucial in triggering and sustaining spondyloarthritis, though its exact mechanisms remain unclear [10]. The Piezo1 channel is a significant discovery in the field of mechanosensitive ion channels. It responds to various mechanical forces and other stimuli, playing a crucial role in the regulation of differentiation, cell proliferation, and function [11]. It is widely distributed across the cardiovascular, pulmonary, urological, skeletal, and immune systems [12]. Existing studies suggest that Piezo1-mediated mechanotransduction enhances pathological bone formation in AS patients via the CaMKII signaling pathway [13]. However, as an immune-mediated inflammatory disease, it remains unclear whether Piezo1 modulates the immune-inflammatory response in AS patients.

Cellular adaptation to physical environmental changes is vital for functional homeostasis [14]. One key mechanism by which cells respond to mechanical stress is the induction of autophagy. As a highly conserved cellular process, autophagy plays a crucial role in maintaining internal homeostasis [15]. Our previous research has identified a closely association between single nucleotide polymorphisms in autophagy-related genes, such as ULK1, IRGM, and ATG16L1, and genetic susceptibility to AS [16–18]. Francesco et al. demonstrated that misfolded HLA-B27 proteins in the intestines of AS patients are linked to the activation of autophagy [19]. Additionally, it has been shown that the number of intracellular autophagosomes increases in response to mechanical stress, and sustained mechanical stress results in continued autophagy activation [20]. Shim et al. demonstrated that trabecular meshwork cells subjected to cyclic mechanical stress also show autophagy activation, accompanied by nuclear accumulation of LC3B [21]. In a recent study, reduced levels of Piezo1 and autophagy were observed in patients and mice with Marfan syndrome (MFS). Piezo1 conditional knockout MFS mice displayed further reductions in autophagy and exacerbation of disease symptoms, while treatment with the Piezo1-specific agonist Yoda1 led to a significant increase in autophagy levels and improved disease symptoms [22]. However, whether Piezo1 regulates autophagy in AS patients remains unclear. In this study, we investigate the specific mechanism by which Piezo1 mediates autophagy to regulate the immune-inflammatory response in AS, providing scientific evidence for a deeper understanding of the pathological mechanisms of AS and potential therapeutic strategies targeting Piezo1.

## Results

### Piezo1 is up-regulated and correlated with autophagy-related genes in AS patients

**Fig. 1:**
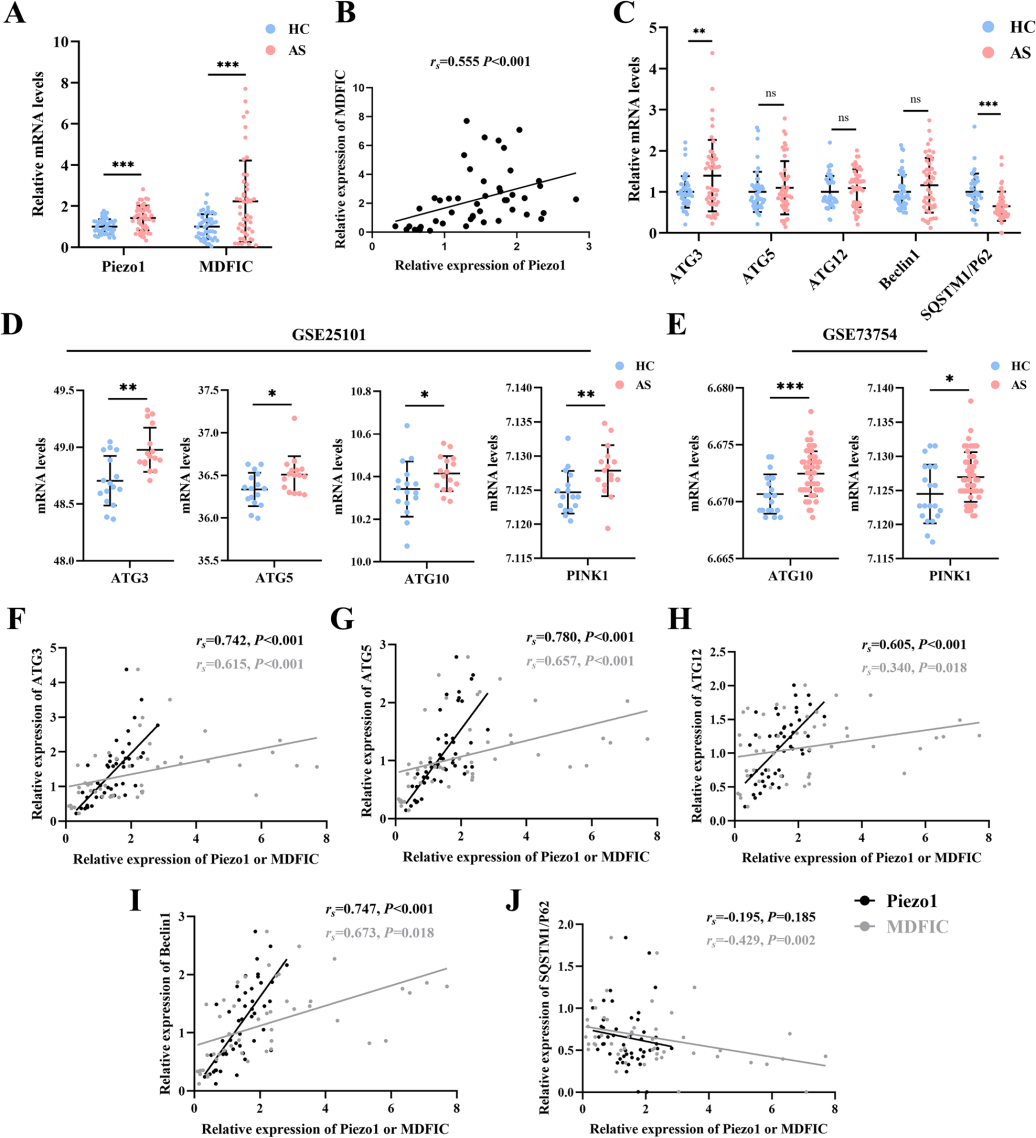
Levels of Piezo1 and autophagy-related genes in AS patients. **A** RT-qPCR analysis of Piezo1 and MDFIC expression levels in PBMCs from AS patients (n = 48) and (n = 48). **B** Correlation analysis between Piezo1 and MDFIC. **C** RT-qPCR analysis of autophagy-related gene expression (ATG3, ATG5, ATG12, Beclin1, and SQSTM1/P62) in PBMCs from AS patients and HC. **D** Relative expression of autophagy-related genes in the GSE25101 (HC, n = 16; AS, n = 16) whole-blood genome sequencing dataset. **E** Relative expression of autophagy-related genes in the GSE73754 (HC, n = 20; AS, n = 52) whole-blood genome sequencing dataset. Correlation analysis of Piezo1 or MDFIC with ATG3 (**F**), ATG5 (**G**), ATG12 (**H**), Beclin1 (**I**) and SQSTM1/P62 (**J**) in AS patients. ^*^P < 0.05, ^**^P < 0.01, ^***^P < 0.001; ns, difference not statistically significant. AS ankylosing spondylitis, HC healthy control, *r*_s_, Spearman rank correlation coefficient.

**Fig. 2:**
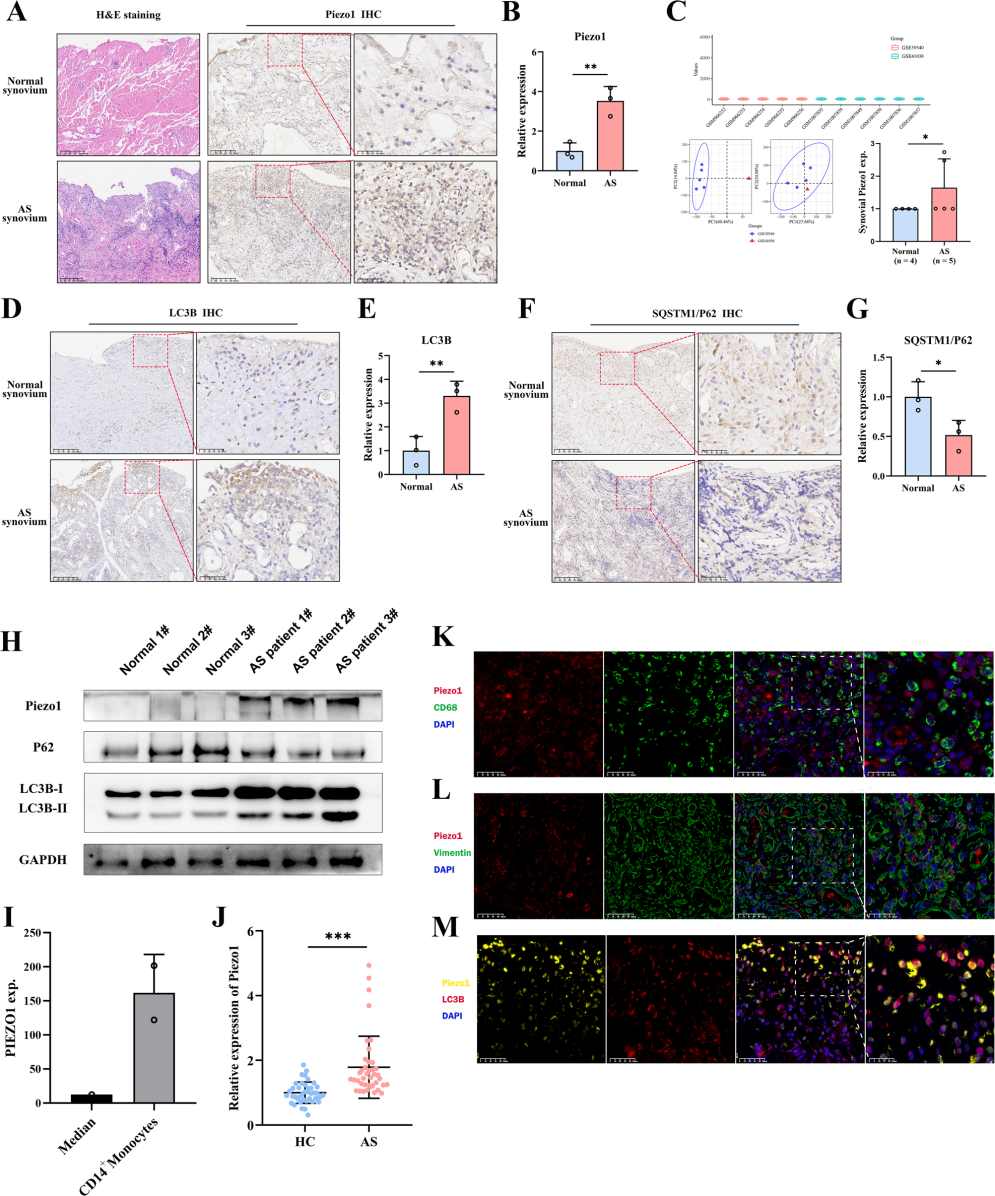
Expression of Piezo1 and autophagy-related proteins in AS patients. **A**, **B** H&E staining of hip synovium from AS patients and fracture controls, and IHC staining with quantitative analysis of Piezo1 expression, n = 3. **C** Relative expression of Piezo1 in the GSE39340 (AS, n = 5) and GSE41038 (HC, n = 2; AS, n = 4) synovial genome sequencing datasets. **D**, **E** IHC staining and quantitative analysis of LC3B expression in hip synovium from AS patients and fracture controls, n = 3. **F**, **G** IHC staining and quantitative analysis of SQSTM1/P62 expression in hip synovium from AS patients and fracture controls, n = 3. **H** Western blotting results of Piezo1, SQSTM1/P62, and LC3B expression in hip joint synovium of AS patients and controls. **I** BioGPS Gene Portal data comparing PIEZO1 expression in human CD14^+^ monocytes relative to median expression levels in other tissues. **J** Relative expression of Piezo1 in CD14⁺ monocytes from AS patients (n = 40) and HCs (n = 40). Double-stained immunofluorescence showing co-localization of Piezo1 with CD68^+^ macrophages (**K**), Vimentin^+^ FLS (**L**) and LC3B (**M**) in the hip synovium of AS patients. ^*^P < 0.05, ^**^P < 0.01, ^***^P < 0.001. AS ankylosing spondylitis, HCs healthy controls, IHC immunohistochemistry.

To explore Piezo1’s role in AS, we used RT-qPCR to quantify its mRNA expression in peripheral blood mononuclear cells (PBMCs) from AS (n = 48) and healthy controls (HC) (n = 48). Additionally, we assessed the expression of MDFIC, an auxiliary subunit of Piezo1, which partially reflects Piezo1 levels [23]. Table S1 presents a summary of the subjects’ clinical characteristics. RT-qPCR analysis showed that Piezo1 and MDFIC expression was upregulated in AS patients compared with HC (*P* < 0.05; Fig. 1A). Correlation analyses revealed a strong positive correlation between Piezo1 and MDFIC expression levels (Fig. 2B). Furthermore, we examined the associations between Piezo1, MDFIC, and key clinical characteristics of AS patients. As shown in Table 1, Piezo1 expression was positively correlated with monocyte count, Bath AS Disease Activity Index (BASDAI), and disease duration. Similarly, MDFIC expression showed positive correlations with erythrocyte sedimentation rate (ESR), Finger-to-Floor Distance (FFD), BASDAI, and AS Disease Activity Score-ESR (ASDAS-ESR). Subgroup analyses indicated that Piezo1 and MDFIC levels were significantly elevated in AS patients with high disease activity (ASDAS-ESR ≥ 2.1, BASDAI ≥ 4) (*P* < 0.05; Tables S2, S3). However, no significant differences in Piezo1 or MDFIC levels were observed based on pharmacological treatment status (TNF-α inhibitors or NSAIDs) (*P* > 0.05; Table S4).

**Table 1:** Correlation between Piezo1 and MDFIC expression levels and clinical indicators in patients with AS.

Indicators	Piezo1		MDFIC	
	*r*_*s*_	*P* value	*r*_*s*_	*P* value
ESR	0.238	0.129	0.342	**0.026**
CRP	-0.012	0.941	0.088	0.596
LYM	-0.164	0.288	-0.245	0.109
MON	0.301	**0.047**	0.267	0.079
FFD	0.265	0.071	0.401	**0.005**
BASDAI	0.364	**0.011**	0.288	**0.047**
BASFI	0.118	0.423	-0.062	0.677
ASDAS-ESR	0.279	0.055	0.402	**0.005**
Disease duration	0.334	**0.020**	-0.120	0.417

*AS* ankylosing spondylitis, *ASDAS* Ankylosing Spondylitis Disease Activity Score, *BASDAI* Bath Ankylosing Spondylitis Disease Activity Index, *BASFI* Bath Ankylosing Spondylitis Functional Index, *CRP* C-reactive protein, *ESR* erythrocyte sedimentation rate, *FFD* Finger-floor distance, *LYM* lymphocyte, *MON* monocyte, *r*_*s*_: Spearman’s correlation coefficient.

*P* values with bold were considered statistically significant differences.

The critical role of autophagy in AS has been well established [19]. RT-qPCR analysis revealed a significant upregulation of ATG3, whereas SQSTM1/P62 was significantly downregulated (*P* < 0.05; Fig. 1C). Although ATG5, ATG12, and Beclin1 levels showed an upward trend in AS compared to HC, the differences were not statistically significant (*P* > 0.05; Fig. 1C). Further correlation analysis between autophagy-related genes and AS disease characteristics revealed a significant positive correlation between ATG3 expression and ESR (Table S5). Additionally, subgroup analyses did not indicate significant differences in autophagy-related gene expression across disease activity indices (*P* > 0.05; Tables S2, S3). However, when stratified by TNF-α inhibitor use, ATG3 and Beclin1 levels were significantly higher in AS patients who were not receiving TNF-α inhibitors compared to those who were (*P* < 0.05; Table S4). In contrast, no significant differences in autophagy-related gene expression were observed in the subgroup analysis based on NSAID use (*P* > 0.05; Table S4). Given the abnormal expression of autophagy-related genes identified in AS patients in this study, we further analyzed whole-blood genome sequencing data from two GEO datasets (GSE25101, GSE73754). The results showed a significant upregulation of autophagy-related genes, including ATG3, ATG5, ATG10, and PINK1, in AS patients compared to HC (Fig. 1D, E). In contrast, no statistically significant differences are observed in the expression of Piezo1, ATG12, Beclin1, and SQSTM1/P62 in the same datasets (Fig. S1).

Furthermore, correlation analysis showed significant positive associations between Piezo1 and MDFIC expression levels with ATG3, ATG5, ATG12, and Beclin1 in AS patients. Moreover, MDFIC exhibited a significant negative correlation with SQSTM1/P62, while Piezo1 showed a negative but non-significant correlation (*P* = 0.185) (Fig. 1F–J). These findings collectively indicate that Piezo1 may influence autophagy and contribute to AS pathogenesis.

### Piezo1 is upregulated in synovial tissue from AS patients and expressed in macrophages

To further investigate the regulatory role of Piezo1 in AS, we collected hip synovial tissues from AS and fracture controls. Baseline demographic characteristics are summarized in Table S6. Hematoxylin-eosin (H&E) staining revealed that synovial tissues from AS patients exhibited characteristic pathological features of chronic inflammation, including synovial thickening, significant inflammatory cell infiltration, and neovascularization, compared to controls (Fig. 2A, left). Immunohistochemical (IHC) staining demonstrated a significant upregulation of Piezo1 expression in the hip joint synovium of AS compared to controls (Fig. 2A, right; Fig. 2B). Furthermore, synovial genome sequencing data from the GEO database (GSE39340, GSE41038) corroborated these findings, showing increased Piezo1 expression in the synovium of AS patients (Fig. 2C). Given the aberrant expression of autophagy-related genes in PBMCs, we further assessed their expression in synovial tissues. IHC staining confirmed a significant upregulation of LC3B expression in the hip synovium of AS compared to controls (Fig. 2D, E), while SQSTM1/P62 expression was significantly downregulated (Fig. 2F, G). Western blotting analysis further confirms that, compared to the control group, AS patients exhibit elevated Piezo1 expression in the hip synovium, along with increased autophagy activity, as evidenced by upregulated LC3B levels and reduced SQSTM1/p62 expression (Fig. 2H).

Population-based correlation analysis showed a significant positive correlation between Piezo1 expression and the number of CD14^+^ monocytes (Table 1). Additionally, the BioGPS database indicated high Piezo1 expression in CD14+ monocytes (Fig. 2I), suggesting that Piezo1 may play a role in CD14^+^ monocytes in AS. To further investigate, we measured Piezo1 mRNA expression in CD14^+^ monocytes from 40 HC and 40 AS patients, with the clinical characteristics of the subjects provided in Table S7. RT-qPCR results showed that Piezo1 expression was significantly upregulated in CD14^+^ monocytes from AS patients compared to HC (Fig. 2J). Correlation analyses between Piezo1 expression and clinical characteristics of AS patients showed significant positive correlations with pillow wall distance (PWD), the Bath AS Functional Index (BASFI), and disease duration (Table S8). These findings suggest that Piezo1 may be involved in the pathogenesis of AS through CD14^+^ monocytes.

The intrasynovial cellular population primarily consists of synovial-like macrophages and FLS. Given that CD14^+^ monocytes are one of the major source of macrophages in synovial tissue [24], and considering the osteogenic differentiation capacity of FLS in AS [25], we performed immunofluorescence (IF) staining. This revealed that Piezo1 was co-expressed with macrophages (CD68^+^) and FLS (Vimentin^+^) in the synovium of AS patients’ hip joints (Fig. 2K, L). Additionally, IF analysis showed co-localization of Piezo1 and LC3B, with double-positive Piezo1⁺/LC3B⁺ staining (Fig. 2M). Collectively, these results suggest that Piezo1 and LC3B may interact in macrophages and FLS, contributing to the regulation of AS pathogenesis.

### Effect of the Piezo1-autophagy axis on monocyte-macrophages in vitro

**Fig. 3:**
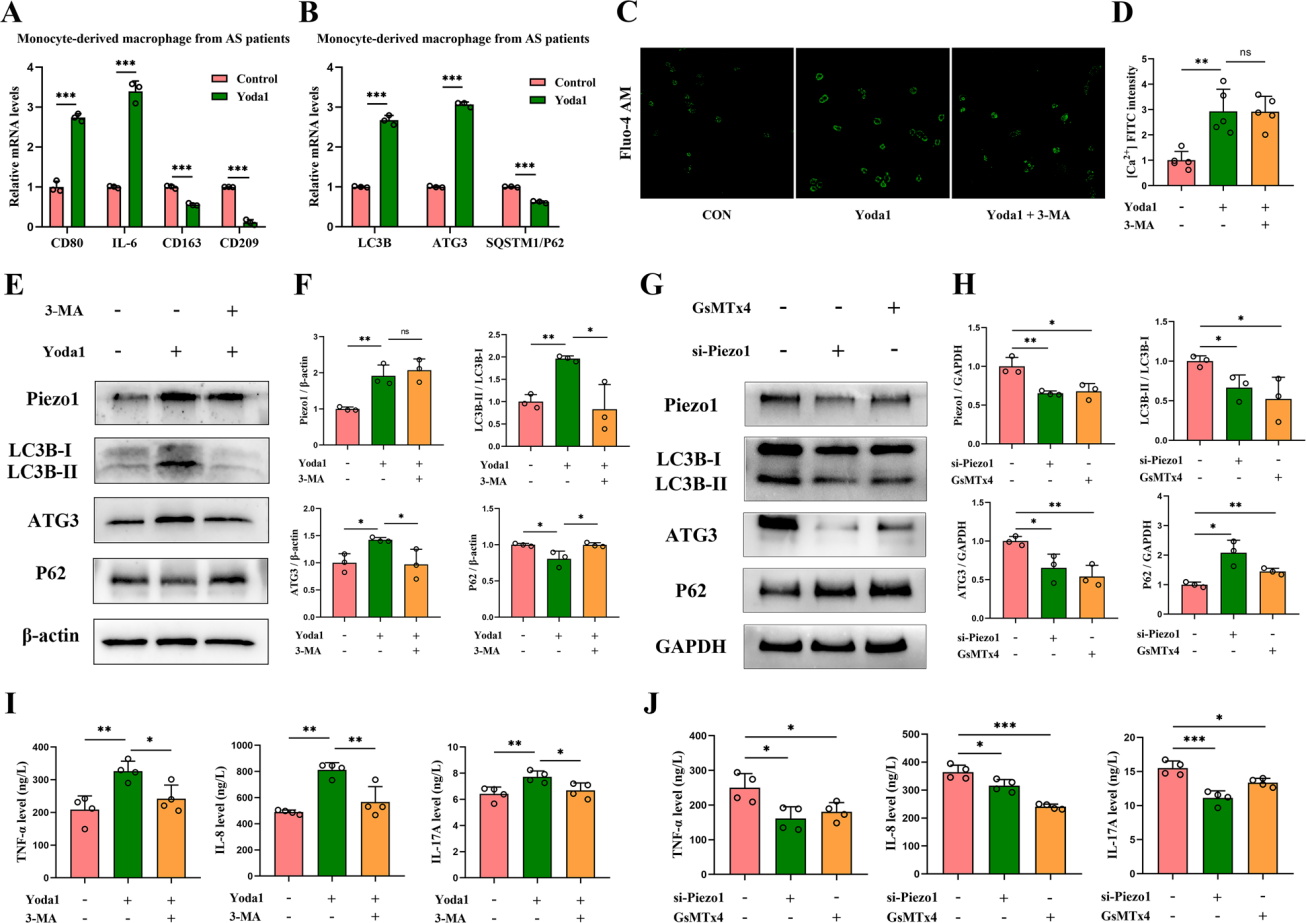
Effects of Piezo1 and autophagy on monocyte-derived macrophages in vitro. **A** Effect of Yoda1-mediated Piezo1 upregulation on primary macrophage polarization (n = 3). **B** Effect of Yoda1-mediated Piezo1 upregulation on autophagy-related gene expression in primary macrophages (n = 3). **C**, **D** Effect of Yoda1 and 3-MA intervention on intracellular Ca^2+^ levels in primary macrophages (n = 5), Scale bar: 10 μm. **E**, **F** Western blot analysis of Piezo1 and autophagy-related protein expression following Yoda1 and 3-MA intervention (n = 3). **G**, **H** Western blot analysis of Piezo1 and autophagy-related protein expression following si-Piezo1 and GsMTx4 intervention (n = 3). **I** ELISA quantification of TNF-α, IL-8, and IL-17A expression in primary macrophages after Yoda1 and 3-MA intervention (n = 4). **J** ELISA quantification of TNF-α, IL-8, and IL-17A expression in primary macrophages after si-Piezo1 and GsMTx4 intervention (n = 4). ^*^P < 0.05, ^**^P < 0.01, ^***^P < 0.001; ns, difference not statistically significant.

Based on the positive correlation between Piezo1 and autophagy in the AS population, we hypothesized that Piezo1 promotes autophagy in AS. To test this, we treated CD14^+^ monocyte-derived macrophages from AS patients with the Piezo1-specific agonist Yoda1. RT-qPCR results showed upregulation of pro-inflammatory markers (M1) and suppression of anti-inflammatory marker (M2) expression in Yoda1-treated monocyte-macrophages (Fig. 3A). Additionally, Yoda1 intervention led to increased autophagy, evidenced by elevated LC3B and ATG3 levels and reduced SQSTM1/P62 levels (Fig. 3B). Fluo-4 AM staining revealed that Yoda1 significantly increased intracellular calcium ion levels in monocyte-macrophages. However, no significant change in calcium ion fluorescence intensity was observed following the addition of the autophagy inhibitor 3-MA (Fig. 3C, D). These findings suggest that the mechanism by which Piezo1 regulates calcium ion influx is independent of autophagy levels.

To further investigate the regulatory role of the Piezo1-autophagy axis in monocyte-macrophages, Western Blotting results showed that Yoda1 treatment significantly increased Piezo1 expression in monocyte-macrophages compared to the control group. This was accompanied by upregulation of autophagy markers LC3B and ATG3 and downregulation of SQSTM1/P62. Further intervention with the autophagy inhibitor 3-MA did not significantly alter Piezo1 expression but decreased autophagy levels, confirming the upstream-downstream regulatory relationship between Piezo1 and autophagy (Fig. 3E, F). ELISA results revealed that Piezo1 elevation promoted the secretion of TNF-α, IL-8, and IL-17A, and further administration of 3-MA reversed the secretion of these inflammatory cytokines (Fig. 3I). To further confirm the role of Piezo1, we performed siRNA-mediated Piezo1 knockdown and Piezo1 inhibitor GsMTx4. Both approaches significantly reduced Piezo1 protein levels and autophagy activation, as evidenced by decreased LC3B and ATG3 and increased SQSTM1/P62 expression (Fig. 3G, H). ELISA further revealed that TNF-α, IL-8, and IL-17A levels were significantly downregulated following both interventions (Fig. 3J). These findings further confirm that Piezo1 promotes inflammatory cytokine secretion in monocyte-macrophages by enhancing autophagy.

### Effect of the Piezo1-autophagy axis on FLS in vitro

**Fig. 4:**
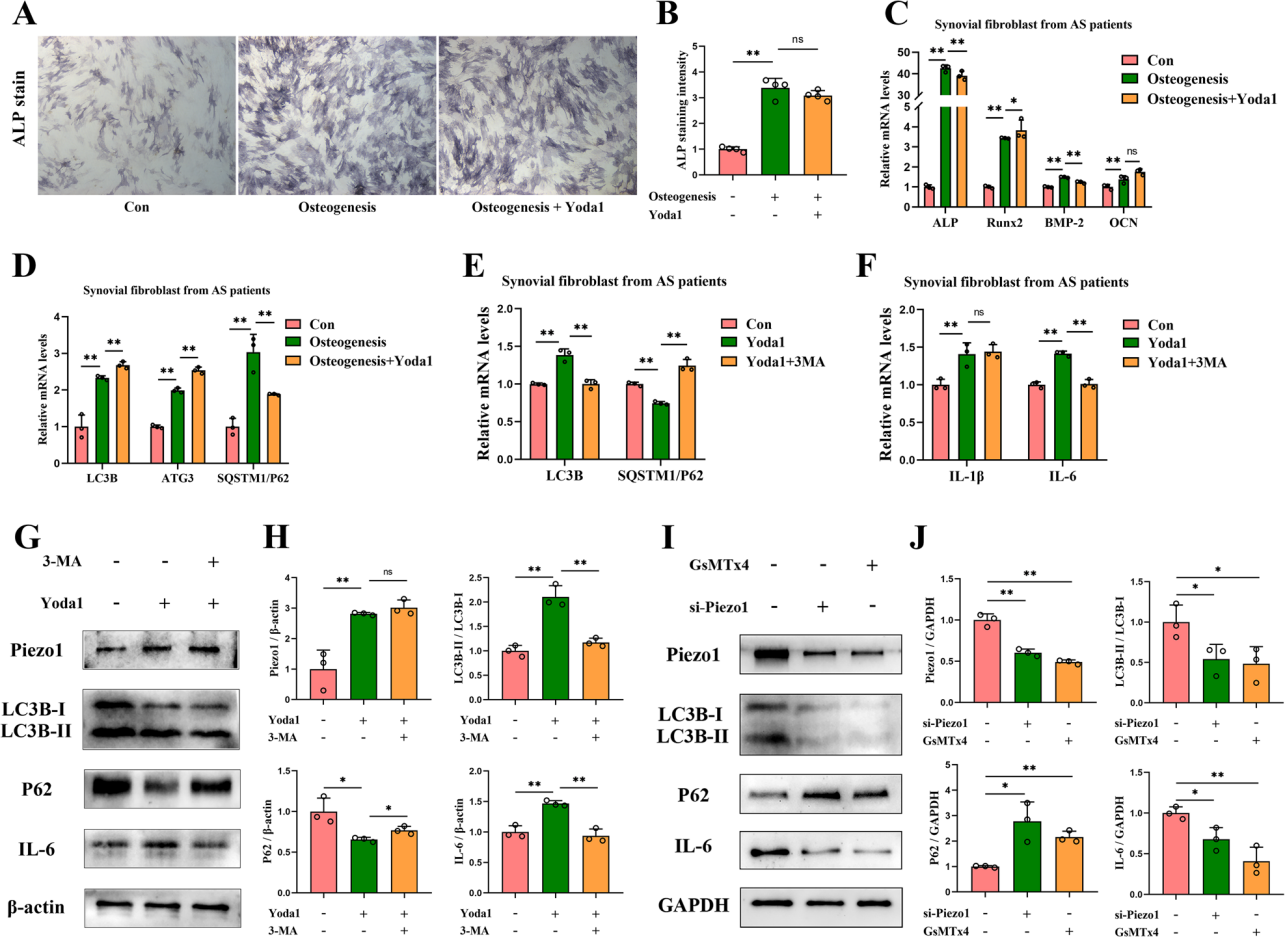
Effects of Piezo1 and autophagy on FLS in vitro. **A**, **B** Effect of Yoda1-mediated Piezo1 upregulation on ALP expression in FLS (n = 4). Scale bar: 10 μm. **C** Effect of Yoda1-mediated Piezo1 upregulation on osteogenic differentiation-related gene expression in FLS (n = 3). **D** Effect of Yoda1-mediated Piezo1 upregulation on autophagy-related gene expression in FLS (n = 3). **E** Expression levels of autophagy-related genes in FLS following Yoda1 and 3-MA interventions (n = 3). **F** Expression levels of inflammation-related genes after Yoda1 and 3-MA intervention (n = 3). **G**, **H** Western blot analysis of autophagy and IL-6 protein expression in FLS following Yoda1 and 3-MA intervention (n = 3). **I**, **J** Western blot analysis of autophagy and IL-6 protein expression in FLS following si-Piezo1 and GsMTx4 intervention (n = 3). ^*^P < 0.05, ^**^P < 0.01^;^ ns difference not statistically significant.

Given that FLS in AS patients showed greater osteogenic differentiation [25], and the accompanying elevated autophagy levels during this process [26], we hypothesized that Piezo1 may promote the osteogenic differentiation of FLS by inducing autophagy. Alkaline phosphatase (ALP) staining revealed a marked increase in ALP expression in FLS following osteogenic induction. However, no further increase in ALP expression was observed following Yoda1 treatment, and a decreasing trend was noted (Fig. 4A, B). RT-qPCR results revealed that, compared to the control group, mRNA expression of ALP, RUNX2, BMP-2, and OCN in osteogenically induced FLS was significantly elevated. However, no further increase was seen after Yoda1 intervention (Fig. 4C). Additionally, autophagy levels were elevated in FLS during osteogenic induction, with further enhancement observed following Yoda1 treatment, as evidenced by upregulation of LC3B and ATG3 and downregulation of SQSTM1/P62 (Fig. 4D). Thus, although FLS osteogenic differentiation was accompanied by elevated autophagy levels, Piezo1 activation did not further promote osteogenic differentiation, despite a significant increase in autophagy levels following Piezo1 intervention.

Given that FLS, as a major contributor and mediator of inflammation, is not only a major producer of IL-6 in inflammatory diseases, but is also capable of inducing the production of various inflammatory factors such as IL-1β [27, 28]. Based on this, we further investigated whether Piezo1-mediated autophagy regulates the expression of inflammatory factors in FLS from AS patients. RT-qPCR results showed that Yoda1 treatment significantly upregulated LC3B expression and downregulated SQSTM1/P62 expression compared to the control group, whereas 3-MA treatment significantly suppressed autophagy levels (Fig. 4E). Additionally, Yoda1 treatment significantly increased the mRNA levels of IL-1β and IL-6, whereas 3-MA treatment only led to a significant downregulation of IL-6 mRNA levels (Fig. 4F). Western blotting further confirmed these results (Fig. 4G, H). In addition, siRNA-mediated knockdown of Piezo1 and treatment with the Piezo1 inhibitor GsMTx4 both reduced Piezo1 and LC3B levels, increased SQSTM1/P62 expression, and suppressed IL-6 production in FLS (Fig. 4I, J). Taken together, these results suggest that Piezo1 modulates IL-6 expression by regulating autophagy in FLS from AS patients.

### Piezo1 inhibitor mitigates spinal arthritis of Proteoglycan-induced arthritis (PGIA) mice

**Fig. 5:**
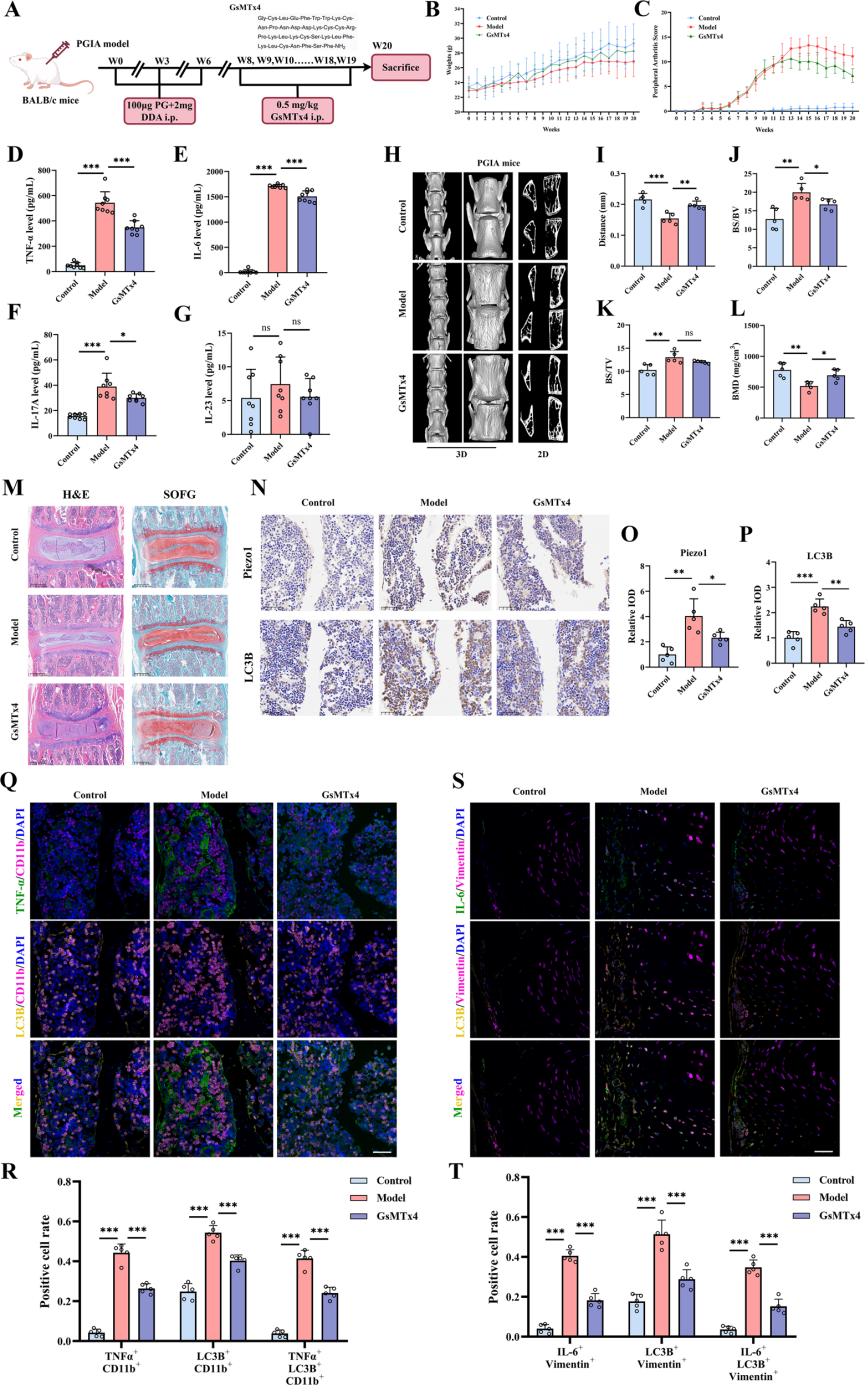
Piezo1 inhibition attenuates spinal arthritis in proteoglycan-induced arthritis (PGIA) mice. **A** GsMTx4 was used in a PGIA model to evaluate the effect of direct Piezo1 inhibition on arthritis progression. **B**, **C** GsMTx4 mitigates body weight loss and reduces the peripheral arthritis index (n = 9 per group at the start of the experiment). **D**–**G** GsMTx4 decreases peripheral blood inflammatory factor levels in 20-week PGIA mice (n = 8 per group; serum samples from one mouse in each group were excluded due to hemolysis). **H** μCT analysis of spinal tissues in the PGIA model, including 2D and 3D reconstructions. Quantification of spinal bone parameters by μCT analysis, including spinal bone spacing (**I**), BS/BV (**J**), BS/TV (**K**), and BMD (**L**) (n = 5 per group, randomly selected due to availability of intact tissue for μCT). **M** H&E and SOFG staining of spinal tissues. **N**, **P** IHC staining demonstrating that GsMTx4 suppresses the upregulation of Piezo1 and LC3B in the bone marrow cavity of PGIA spines (n = 5). **Q**, **R** IF analysis and quantification of LC3B and TNF-α in CD11b^+^ macrophages within the bone marrow cavity (n = 5), scale bar: 50 µm. **S**, **T** IF analysis and quantification of LC3B and IL-6 in Vimentin^+^ FLS located within the intervertebral tissue region (n = 5), scale bar: 50 µm. ^*^P < 0.05, ^**^P < 0.01, ^***^P < 0.001; ns, difference not statistically significant. BMD bone mineral density, BS bone surface, BV bone volume, SOFG Safranin O-Fast Green, TV tissue volume.

PGIA is a well-established model of spinal arthritis (Fig. 5A). This study evaluated the therapeutic effect of the Piezo1 inhibitor GsMTx4. Briefly, GsMTx4 treatment improved body weight (Fig. 5B, Table S9) and reduced the peripheral arthritis index (Fig. 5C, Table S10) in PGIA mice. ELISA assays showed that, by week 20, GsMTx4 significantly decreased serum levels of TNF-α, IL-6, and IL-17A compared to the model group (Fig. 5D–F); however, IL-23 levels did not show significant changes (Fig. 5G). Micro-CT results indicated that GsMTx4 improved spinal bone surface roughness and reduced joint fusion symptoms in PGIA mice (Fig. 5H, I). It also decreased the bone surface area to volume ratio (BS/BV) (Fig. 5J), while showing a trend towards a lower, but not statistically significant, difference in the bone surface area to tissue volume ratio (BS/TV) (Fig. 5K). Furthermore, GsMTx4 treatment mitigated the reduction in spinal bone mineral density (BMD) (Fig. 5L). H&E and Safranin O-Fast Green (SOFG) staining revealed partial recovery of spinal nucleus pulposus size and reduced fibrosis and inflammatory cell infiltration in the GsMTx4 treatment group (Fig. 5M). IHC results demonstrated that GsMTx4 suppressed the abnormal increase of Piezo1 and LC3B in the spinal marrow cavity of PGIA mice (Fig. 5N, P). IF analysis further suggested that the therapeutic effects of GsMTx4 may involve modulation of autophagy and inflammatory responses in specific cellular populations. In the spinal marrow cavity, CD11b^+^ macrophages exhibited increased TNF-α and LC3B levels in PGIA mice, which were reduced following GsMTx4 treatment (Fig. 5Q, R). Similarly, in the intervertebral tissue region, Vimentin⁺ FLS showed elevated IL-6 and LC3B levels in the PGIA model, and these were decreased upon GsMTx4 intervention (Fig. 5S, T). Taken together, these results suggest that GsMTx4 treatment significantly inhibited the development of spondyloarthritis in the PGIA model, including inflammation, autophagy activation in macrophages and FLS, and bone structural changes.

## Discussion

Piezo1 is a non-selective cation channel that plays a crucial role in the cardiovascular, pulmonary, tumor, and immune systems [12]. Previous studies have demonstrated that upregulation of Piezo1 promotes new bone formation in the tibia of patients with AS [13]. Although heterotopic ossification is a prominent pathological feature of AS, the immune-inflammatory response-particularly in the early stages-also plays a pivotal role in disease progression [29]. Given Piezo1’s regulatory role in immune cells [30] and the critical involvement of various immune cells in AS [31], we hypothesized that Piezo1 may be an important mediator of the immune-inflammatory response in AS. In this study, we found that Piezo1 and MDFIC were significantly upregulated in AS patients and were strongly correlated with clinical parameters such as ESR, finger-ground distance, disease duration, BASDAI, and ASDAS-ESR scores. Additionally, we observed significant upregulation of Piezo1 protein levels in synovial tissues of AS patients, a finding consistent with data from the GEO database (GSE25101 and GSE73754). Similarly, Piezo1 protein levels were significantly elevated in the spinal tissues of proteoglycan-induced AS mice.

Autophagy plays a key role in modulating immune-inflammatory responses [15]. Mechanical stress has been shown to be closely associated with autophagy through mechanisms involving integrin adhesion receptors, cytoskeleton, and mechanosensitive ion channels [32]. Piezo1, the largest mechanosensitive ion channel, is crucial in the immune-inflammatory response and closely linked to the pathogenesis of AS. In this study, we examined the expression levels of autophagy-related genes in PBMCs from AS patients. We found that ATG3 expression was significantly upregulated, SQSTM1/P62 was downregulated, while ATG5, ATG12, and Beclin1 showed a non-significant trend toward upregulation. We hypothesized that these findings may be influenced by pharmacological treatment, as nearly two-thirds of the study population were receiving biologic therapies. Supporting this, subgroup analysis revealed that the expression of autophagy-related genes (e.g., ATG3 and Beclin1) was significantly higher in AS patients not treated with TNF-α inhibitors. To validate this hypothesis, we further analyzed whole blood genome sequencing data from two AS case-control studies (GSE25101 and GSE73754) in the GEO database, which showed significant upregulation of autophagy-related genes, in AS. Importantly, the AS populations in these studies did not receive biologic treatments [33, 34]. Additionally, IHC staining of synovial tissue from the hip further supports the elevated levels of autophagy in AS patients. Notably, mRNA and protein levels of autophagy-related genes (e.g., LC3B, ATG5, ATG12) have also been reported to be upregulated in the intestinal tissues of AS patients, particularly in monocytes [19]. However, this view is challenged by Neerinckx et al., whose study found no significant activation of autophagy (at the mRNA level) in AS patients [35]. Tissue specificity may account for some of these discrepancies; additionally, the small sample size and the selection of knee synovial tissue in their study may not adequately represent the pathology of AS. In conclusion, the present study confirms that Piezo1 plays a significant role in the development of AS by mediating autophagy, as demonstrated by population correlation analysis and tissue fluorescence co-localization.

The role of the mechanosensitive ion channel Piezo1 in regulating autophagy remains controversial, with limited studies addressing this relationship in AS. Existing literature suggests that Piezo1 induces autophagy in certain neurological diseases. For instance, Yue et al. demonstrated that silencing Piezo1 alleviated disease symptoms and reduced autophagy in stroke rats, while autophagy levels were significantly elevated under oxygen-glucose deprivation conditions in HT22 cells, with silencing Piezo1 reversing this effect [36]. In cardiovascular diseases, Piezo1’s role in autophagy regulation has also been established. Yang et al. [22]. reported decreased levels of Piezo1 and autophagy in Marfan syndrome (MFS) patients and mice, with further reduction of autophagy in Piezo1 conditional knockout MFS mice, which exacerbated disease symptoms. In vitro experiments also confirmed the promoting effect of Piezo1 on autophagy. In osteoblasts, Piezo1 activation induced by matrix mechanical stiffness upregulated autophagy through the AMPK-ULK1 axis, promoting osteogenic differentiation [37]. However, other study present conflicting perspectives. Shi et al. reported elevated Piezo1 expression in disc degeneration and demonstrated in nucleus pulposus cells that excessive mechanical stress-induced upregulation of Piezo1 promotes apoptosis and pro-inflammatory factor release, while suppressing autophagy [38]. These contradictory findings may be attributed to two factors: first, the complex and context-dependent functions of autophagy in different cells and tissues, which may even exhibit dual roles [39]; second, the high-intensity mechanical stress used in Shi et al.‘s study (1 Hz, 15% compressive strength) might not be applicable to other diseases or cell types, where mechanical forces are not as extreme [40, 41]. Notably, in chondrocytes, normal-intensity mechanical forces promote autophagy, whereas excessive forces (4000 μstrain) inhibit it [42]. In summary, Piezo1’s regulation of autophagy appears to be cell-specific and context-dependent, with its precise mechanism in AS remaining to be further investigated.

In the present study, we observed significant upregulation of Piezo1 and autophagy levels in both AS patients and mice. In vitro experiments demonstrated that Piezo1 induced M1-type polarization in monocyte-macrophages and elevated autophagy levels, which subsequently promoted the secretion of inflammatory factors, including TNF-α, IL-8, and IL-17A. In FLS, autophagy levels were significantly elevated during osteogenic induction, consistent with the findings of Yang et al. However, after Yoda1 intervention, no significant increase in osteogenic differentiation was observed, despite the further elevation of autophagy. We propose two potential explanations for this observation. First, the osteogenic induction medium contains dexamethasone, L-ascorbic acid, and β-glycerophosphate. L-ascorbic acid regulates histone demethylation and promotes TET-mediated hydroxymethylation, thereby facilitating osteogenic differentiation [43]. Notably, L-ascorbic acid is also a specific inhibitor of Cav3.2 channels [44], which may interfere with calcium ion influx and intracellular calcium concentrations. In contrast, Piezo1 activation mediates inward calcium ion flow [12], potentially creating a partial antagonistic effect between the two pathways, thus impairing osteogenic differentiation. Second, we suggest that Piezo1 does not directly induce osteogenic differentiation in FLS from AS patients. While FLS in AS patients are known to possess enhanced osteogenic differentiation capacity [25], the prevailing view is that mesenchymal stem cells (MSCs) and osteoblasts are the primary effector cells in AS [45, 46]. Additionally, Chen et al. [13]. demonstrated that Piezo1 mediates ectopic ossification in AS primarily through osteoblasts and chondrocytes. However, FLS, as crucial regulators of inflammation [28], likely play an important role in the inflammatory processes of AS. Previous studies have shown that Piezo1 activation in FLS increases IL-6 secretion at both the mRNA and protein levels, although the exact regulatory mechanism remains unclear [47]. Our study further revealed that Piezo1 promotes IL-6 secretion by mediating autophagy elevation, suggesting that autophagy may serve as a key intermediary in this process. These findings were supported by animal experiments, where increased autophagy and inflammatory factors were observed in AS mice, particularly within macrophages and FLS. Treatment with the Piezo1 inhibitor GsMTx4 significantly reduced these changes, offering osteoprotective effects on the spine. This result not only confirms Piezo1’s role in regulating autophagy and inflammation in AS but also suggests that inhibition of Piezo1 could be a promising strategy for managing AS-related symptoms by modulating autophagy levels.

In addition to its regulatory roles in inflammation and autophagy, Piezo1 may also be involved in the osteoimmunological processes underlying AS. The interaction between bone cells—including mesenchymal stem cells (MSCs), osteoblasts, and osteoclasts—and immune cells such as T and B lymphocytes has been increasingly recognized as a critical contributor to pathological bone remodeling in AS [48]. Recent studies have demonstrated that Piezo1 regulates osteoblast differentiation via mechanical signal transduction and the AMPK-autophagy pathway in a matrix stiffness-dependent manner, highlighting a mechanotransduction–autophagy axis in skeletal biology [37]. Moreover, Piezo1 has been shown to modulate bone metabolism by mediating MSCs-Th17 cell crosstalk through glycolytic reprogramming, offering novel insights into its role in immune–bone interactions under inflammatory conditions [49]. These findings suggest that Piezo1-mediated autophagy may serve as a central node linking mechanical cues, immune responses, and bone remodeling, although further in vivo and mechanistic studies are needed to substantiate this hypothesis.

While this study provides valuable insights, it also has several limitations. First, the case-control participants were drawn from a single center and matched only by age and gender, without accounting for other potential confounders, which may introduce selection bias. Therefore, further validation in a multicenter, large-sample study is needed to enhance the reliability and generalizability of the findings. Second, although the present study extensively examined the role of Piezo1 in monocyte-macrophages and FLS in AS patients, it did not explore the interactions between these two cell types, which are crucial to the complex pathogenesis of AS. Future research should focus on intercellular interactions to better understand their role in AS. Finally, while the in vivo function of Piezo1 was validated in the AS mouse model, the systemic administration of GsMTx4 limited its ability to specifically target macrophages or FLS, which may have affected the accuracy and impact of the results. It is worth noting that the choice of animal models in AS research remains controversial, with the proteoglycan-induced AS model typically constructed in BALB/c mice, whereas gene knockout models in specific target cells are typically constructed using C57BL/6 J mice. This genetic background incompatibility imposes a key limitation, preventing the use of tissue-specific Piezo1 knockout strategies in synovial-like macrophages or FLS within the PGIA model. Over the past decades, several theories have been proposed regarding the origin of AS, including the synovial, entheseal, and bone marrow origins. Recent evidence highlights the bone marrow as a central site where local immune activation, inflammation, and osteo-metabolic activity converge to drive pathological new bone formation [50]. Based on this concept, we believe that myeloid cell–specific deletion of Piezo1 would provide a more informative strategy to dissect its role in AS pathogenesis. However, due to genetic background constraints, such experiments could not be implemented at this stage, and their future application in alternative arthritis models, such as collagen-induced arthritis (CIA), may yield more definitive mechanistic insights.

In this study, we report for the first time the role of Piezo1-mediated autophagy in the immune-inflammatory response in AS. Case-control studies revealed that Piezo1 expression was significantly elevated in AS and strongly correlated with clinical indicators, including disease activity and duration, as well as with autophagy levels. Mechanistically, Piezo1 induces autophagy activation and increases inflammatory factor secretion in monocyte-macrophages and FLS. However, inhibition of Piezo1 prevented excessive autophagy activation in vivo, attenuated the inflammatory response, and exerted a protective effect on spinal bone tissue in AS mice. In conclusion, these findings provide a new theoretical framework for understanding the pathological mechanisms of AS and suggest a potential research direction for developing targeted therapeutic strategies against Piezo1.

## Materials and methods

Additional detailed information is provided in the supplementary material.

### Patient and control recruitment

The Ethics Committee of Anhui Medical University approved the procedures for this study (No. 83230287). The diagnostic criteria for AS patients were based on the 1984 revised New York criteria from the American College of Rheumatology. The sample size was determined based on preliminary experiment data showing mean Piezo1 expression levels of 1.452 (SD = 0.404) in AS patients and 1.000 (SD = 0.340) in controls, yielding an estimated effect size (Cohen’s d) of approximately 1.27. With α = 0.05 and 90% power, at least 32 subjects per group were needed. A total of 88 AS patients and 88 age- and sex-matched HC were recruited, with written informed consent obtained from all participants. Researchers collected 5 mL of peripheral blood samples, along with demographic and clinical data. Additionally, 3 AS patients and 3 non-AS patients with fractures were recruited, and hip synovial samples were obtained during hip replacement surgery.

### Cell isolation

Peripheral blood was collected from AS patients and healthy controls. PBMCs were isolated via Ficoll-Hypaque density gradient centrifugation. CD14^+^ monocytes were then extracted using CD14 microbeads (BD Bioscience).

### Cell culture

CD14^+^ monocytes from AS patients were resuspended in RPMI 1640 complete medium with 50 ng/mL M-CSF (Thermo) and seeded into 6-well plates. M0 macrophages were obtained after 7 days of culture.

FLS were isolated from the synovial tissues of AS patients. Briefly, hip joint synovial tissues were cut into 1 mm^3^ pieces under aseptic conditions, placed evenly on the surface of T75 culture flasks, and cultured in DMEM/F12 medium (20% FBS). The culture flasks were positioned at an angle in the incubator until the tissue blocks adhered, after which the flasks were placed flat for further culture. After 24 h, the medium was replaced with DMEM/F12 (10% FBS), and subsequently changed every three days. The third passage of synovial fibroblasts was used for subsequent experiments, and the purity of FLS was confirmed by Vimentin staining.

### Cell treatments and transfection

Osteogenic differentiation in FLS was induced by replacing the regular medium with DMEM/F12 complete medium supplemented with dexamethasone (10 nM), vitamin C (0.05 mg/mL), and β-glycerophosphate (10 mM). The medium was refreshed every three days.

To evaluate the effects of Yoda1 and 3-MA on monocyte-macrophages and FLS, Yoda1 was administered at 10 μM and 3-MA at 5 mM in monocyte-macrophages, while in FLS, Yoda1 was applied at 0.5 μM and 3-MA at 0.5 mM. For Piezo1 inhibition, GsMTx4 was applied at a concentration of 0.5 μM in both monocyte-macrophages and FLS. These concentrations were determined based on CCK-8 assay results or literature references [51, 52].

For siRNA transfection, three siRNA sequences (si-Piezo1-P1, si-Piezo1-P2, and si-Piezo1-P3) were designed to downregulate Piezo1 expression (sequences are listed in Table S12). Monocyte-derived macrophages and FLS were transfected with si-Piezo1 using jetPRIME transfection reagent (Polyplus, France) according to the manufacturer’s instructions. Among them, si-Piezo1-P3 exhibited the highest silencing efficiency and was selected for subsequent experiments. The knockdown efficiency was validated at the mRNA level (Fig. S3).

### Animal

The mice used in this study were 12 weeks inbred female BALB/c mice, each weighing between 22 and 24 g. The mice were maintained on a 12-h light/dark cycle and had unrestricted access to food and water. All husbandry and experimental protocols were approved by the Laboratory Animal Ethics Committee of Anhui Medical University (LLSC20220653). The mice were randomly assigned to three groups: the control group, the PGIA model group, and the GsMTx4 treatment group, with 9 mice in each group.

The PGIA model was established using methods described in previous studies [53, 54]. Briefly, at week 0, mice were injected intraperitoneally with a mixture of 100 µg proteoglycan (PG) (Sigma) and 2 mg dioctadecyldimethylammonium bromide (DDA) (Sigma). The same dose of the antigen mixture was administered as booster injections at weeks 3 and 6. Body weight and arthritis scores were monitored and recorded weekly following the injections. For Piezo1 inhibition, mice received weekly intraperitoneal injections of GsMTx4 (0.5 mg/kg) (MCE) or saline as a negative control, starting at week 8. At week 20, mice were anesthetized and euthanized. At the beginning of the experiment, nine mice were included in each group. Due to hemolysis during serum collection, only eight serum samples per group were included in the ELISA analysis. In the later stages of the experiment, because of tissue integrity and processing limitations, five mice per group were randomly selected for micro-CT scanning and IHC staining. Arthritis severity was independently assessed by two blinded experimenters based on swelling, redness, stiffness, and deformity of the paw joints. The arthritis score for each mouse was the sum of scores from all four limbs. The scoring criteria were as follows: 0 = no redness or swelling; 1 = redness and swelling of one toe; 2 = redness and swelling of more than one toe; 3 = stiffness with loss of flexion/extension and reduced function; 4 = deformity and stiffness of the ankle joint.

### Statistical analysis

Statistical analyses were conducted using SPSS 25.0. Normally distributed data were expressed as mean ± SD and compared between two groups using the *t* test. Non-normally distributed data were presented as median (*P*_*25*_*, P*_*75*_) and analyzed with the Mann–Whitney U or Kruskal-Wallis H test. Categorical variables were reported as frequencies and percentages, with differences between groups evaluated using the chi-square. Spearman correlation tests were used to assess correlations. All tests were two-sided, and *P* < 0.05 was considered statistically significant.

## Supplementary information

SUPPLEMENTAL MATERIAL

Original western blots

## Data Availability

The data supporting this study are available from the corresponding author upon reasonable request.

## Patient and public involvement

Patients and/or the public were not involved in the design, or conduct, or reporting, or dissemination plans of this research.
