# Supplementary material for: Piezo1-mediated autophagy promotes immune-inflammatory responses in ankylosing spondylitis
Source: Cell Death Dis. 2026 Jan 8;17(1):12. doi: 10.1038/s41419-025-08230-7 (PMC12783605; doi:10.1038/s41419-025-08230-7)
Supplement: Supplementary file 1 — SUPPLEMENTAL MATERIAL [file 41419_2025_8230_MOESM1_ESM.docx]

**Piezo1-mediated autophagy promotes immune-inflammatory responses in ankylosing spondylitis**

**Supplementary material**

Table S1 General condition and clinical indicators of study objects

|  | AS (n = 48) | HC (n = 48) | t/ꭓ^2^ | *P* value |
| --- | --- | --- | --- | --- |
| Demographic characteristics |  |  |  |  |
| Age (year) | 34.94 ± 6.31 | 36.04 ± 7.85 | -0.760 | 0.449 |
| Gender (male, %) | 37 (77.1%) | 37 (77.1%) | 0.000 | 1.000 |
| Clinical characteristics |  |  |  |  |
| ESR (mm/h) | 5.50 (3.00-18.25) |  |  |  |
| CRP (mg/L) | 3.30 (1.40-10.90) |  |  |  |
| LYM (×10^9^/L) | 2.06 (1.72-2.53) |  |  |  |
| MON(×10^9^/L) | 0.49 (0.38-0.62) |  |  |  |
| FFD (cm) | 13.00 (0.00-30.00) |  |  |  |
| PWD (cm) | 0.00 (0.00-3.00) |  |  |  |
| Chest expansion (cm) | 3.00 (2.00-4.00) |  |  |  |
| Schober test (cm) | 6.00 (4.00-8.00) |  |  |  |
| BASFI | 0.70 (0.00-2.08) |  |  |  |
| BASDAI | 2.15 (1.00-3.90) |  |  |  |
| ASDAS-ESR | 1.69 (1.09-2.48) |  |  |  |
| Disease duration (month) | 60.00 (18.00-114.00) |  |  |  |
| Treatment (n, %) |  |  |  |  |
| NSAIDs | 25 (52.1%) |  |  |  |
| TNF-α inhibitor | 31 (64.6%) |  |  |  |

Note: AS, ankylosing spondylitis; ASDAS, Ankylosing Spondylitis Disease Activity Score; BASDAI, Bath Ankylosing Spondylitis Disease Activity Index; BASFI, Bath Ankylosing Spondylitis Functional Index; CRP, C-reactive protein; ESR, erythrocyte sedimentation rate; FFD, Finger-floor distance; HC, healthy control; LYM, lymphocyte; MON, monocyte; NSAIDs, nonsteroidal anti-inflammatory drugs; PWD, pillow wall distance. Data were presented as mean ± SD or median (*P_25_, P_75_*).

Table S2 Expression levels of Piezo1, MDFIC, and autophagy-related genes in AS patients: A subgroup analysis based on BASDAI scores.

|  | BASDAI < 4  (n = 35） | BASDAI ≥ 4  (n = 13） | *Z* | *P* |
| --- | --- | --- | --- | --- |
| Piezo1 | 1.306 (0.799, 1.748) | 1.835 (1.390, 2.023) | -2.285 | **0.022** |
| MDFIC | 1.361 (0.408, 2.576) | 2.212 (1.382, 4.803) | -1.926 | 0.054 |
| ATG3 | 1.050 (0.702, 1.693) | 1.802 (0.843, 2.294) | -1.717 | 0.086 |
| ATG5 | 0.894 (0.619, 1.374) | 1.041 (0.833, 1.711) | -1.149 | 0.251 |
| ATG12 | 1.122 (0.662, 1.420) | 1.068 (0.725, 1.492) | -0.302 | 0.763 |
| Beclin 1 | 0.873 (0.545, 1.563) | 1.333 (0.841, 1.861) | -1.462 | 0.144 |
| SQSTM1/P62 | 0.578 (0.466, 0.864) | 0.500 (0.376, 0.743) | -1.032 | 0.302 |

Note: Data are presented as median (*P_25_, P_75_*). *P* values with bold were considered statistically significant differences. BASDAI, Bath Ankylosing Spondylitis Disease Activity Index.

Table S3 Expression levels of Piezo1, MDFIC, and autophagy-related genes in AS patients: A subgroup analysis based on ASDAS-ESR scores.

|  | ASDAS-ESR < 2.1  (n = 32） | ASDAS-ESR ≥ 2.1  (n = 16） | *Z* | *P* |
| --- | --- | --- | --- | --- |
| Piezo1 | 1.377 (0.819, 1.702) | 1.791 (1.120, 2.129) | -1.695 | 0.090 |
| MDFIC | 1.091 (0.408, 2.558) | 2.346 (1.429, 4.090) | -2.242 | **0.025** |
| ATG3 | 1.068 (0.699, 1.711) | 1.653 (0.825, 2.448) | -1.564 | 0.118 |
| ATG5 | 0.952 (0.625, 1.360) | 0.903 (0.730, 1.497) | -0.634 | 0.526 |
| ATG12 | 1.179 (0.652, 1.412) | 1.083 (0.696, 1.471) | -0.219 | 0.827 |
| Beclin 1 | 1.096 (0.404, 1.557) | 1.231 (0.830, 1.843) | -1.422 | 0.155 |
| SQSTM1/P62 | 0.657 (0.484, 0.865) | 0.479 (0.365, 0.653) | -1.826 | 0.068 |

Note: Data are presented as median (*P_25_, P_75_*). *P* values with bold were considered statistically significant differences. ASDAS, Ankylosing Spondylitis Disease Activity Score; ESR, erythrocyte sedimentation rate.

Table S4 Expression levels of Piezo1, MDFIC, and autophagy-related genes in AS patients: Subgroup analysis based on the use of TNF-α inhibitors or NSAIDs.

|  | TNF-α inhibitor  (n = 31） | No TNF-α inhibitor  (n = 17） | *Z* | *P* | NSAIDs  (n = 25） | No NSAIDs (n = 23） | *Z* | *P* |
| --- | --- | --- | --- | --- | --- | --- | --- | --- |
| Piezo1 | 1.390(0.662,1.772) | 1.553(0.908,2.308) | -1.067 | 0.286 | 1.297(0.735,1.804) | 1.553(1.098,1.912) | -1.465 | 0.143 |
| MDFIC | 1.324(0.408,2.819) | 2.338(1.382,3.368) | -1.520 | 0.129 | 1.630(0.408,2.885) | 1.952(0.911,3.042) | -0.702 | 0.483 |
| ATG3 | 1.035(0.702,1.658) | 1.729(0.551,0.933) | -2.102 | **0.036** | 1.569(0.690,1.932) | 1.050(0.753,1.693) | -0.268 | 0.788 |
| ATG5 | 0.913(0.558,1.318) | 1.101(0.744,2.112) | -1.423 | 0.155 | 0.938(0.585,1.706) | 0.913(0.773,1.309) | -0.144 | 0.885 |
| ATG12 | 1.122(0.667,1.371) | 1.099(0.640,1.576) | -0.140 | 0.889 | 1.122(0.565,1.592) | 1.099(0.695,1.306) | -0.062 | 0.951 |
| Beclin 1 | 0.873(0.508,1.458) | 1.489(0.770,1.992) | -2.221 | **0.026** | 0.982(0.433,1.833) | 1.235(0.699,1.458) | -0.062 | 0.951 |
| SQSTM1/P62 | 0.574(0.426,0.864) | 0.578(0.469,0.707) | -0.162 | 0.872 | 0.578(0.454,0.895) | 0.574(0.426,0.752) | -0.795 | 0.427 |

Note: Data are presented as median (*P_25_, P_75_*). *P* values with bold were considered statistically significant differences. NSAIDs, non-steroidal anti-inflammatory drugs.

Table S5 Correlation between autophagy-related genes expression levels and clinical indicators in patients with AS

| Clinical characteristic | ATG3 | |  | ATG5 | |  | ATG12 | |  | Beclin 1 | |  | SQSTM1/P62 | |
| --- | --- | --- | --- | --- | --- | --- | --- | --- | --- | --- | --- | --- | --- | --- |
|  | *r_s_* | *P* |  | *r_s_* | *P* |  | *r_s_* | *P* |  | *r_s_* | *P* |  | *r_s_* | *P* |
| ESR | 0.320 | **0.039** |  | 0.142 | 0.368 |  | -0.096 | 0.546 |  | 0.253 | 0.106 |  | -0.205 | 0.192 |
| CRP | 0.090 | 0.585 |  | -0.043 | 0.795 |  | -0.153 | 0.352 |  | 0.129 | 0.434 |  | 0.035 | 0.833 |
| LYM | -0.408 | **0.006** |  | -0.249 | 0.104 |  | -0.081 | 0.603 |  | -0.275 | 0.071 |  | -0.104 | 0.502 |
| MON | 0.338 | **0.025** |  | 0.158 | 0.305 |  | -0.033 | 0.833 |  | 0.272 | 0.074 |  | 0.142 | 0.359 |
| BASDAI | 0.166 | 0.259 |  | 0.140 | 0.343 |  | -0.051 | 0.730 |  | 0.152 | 0.303 |  | -0.163 | 0.269 |
| BASFI | -0.025 | 0.864 |  | 0.093 | 0.531 |  | -0.038 | 0.800 |  | 0.000 | 1.000 |  | -0.055 | 0.710 |
| ASDAS | 0.218 | 0.136 |  | 0.151 | 0.306 |  | -0.052 | 0.724 |  | 0.242 | 0.098 |  | -0.085 | 0.566 |
| Disease duration | 0.059 | 0.691 |  | 0.125 | 0.396 |  | 0.001 | 0.997 |  | 0.122 | 0.407 |  | 0.153 | 0.300 |

Note: *r_s_*: Spearman's correlation coefficient; *P* values with bold were considered statistically significant differences. AS, ankylosing spondylitis; ASDAS, Ankylosing Spondylitis Disease Activity Score; BASDAI, Bath Ankylosing Spondylitis Disease Activity Index; BASFI, Bath Ankylosing Spondylitis Functional Index; CRP, C-reactive protein; ESR, erythrocyte sedimentation rate; LYM, lymphocyte; MON, monocyte.

Table S6 Baseline population characteristics

|  | Age | Gender | Type of surgery |
| --- | --- | --- | --- |
| AS1 | 65 | Female | Hip replacement |
| AS2 | 36 | Male | Hip replacement |
| AS3 | 53 | Male | Hip replacement |
| CON1 | 42 | Male | Hip replacement |
| CON2 | 72 | Female | Hip replacement |
| CON3 | 76 | Male | Hip replacement |

Table S7 General condition and clinical indicators of study objects

|  | AS (n = 40) | HC (n = 40) | t/ꭓ^2^ | *P* value |
| --- | --- | --- | --- | --- |
| Demographic characteristics |  |  |  |  |
| Age (year) | 35.54 ± 11.37 | 33.03 ± 7.20 | 1.128 | 0.263 |
| Gender (male, %) | 32 (80.0%) | 33 (82.5%) | 0.000 | 1.000 |
| Clinical characteristics |  |  |  |  |
| ESR (mm/h) | 6.00 (2.50-11.50) |  |  |  |
| CRP (mg/L) | 2.50 (1.07-5.30) |  |  |  |
| Global pain | 3.00 (0.25-3.75) |  |  |  |
| Night pain | 0.00 (0.00-3.00) |  |  |  |
| FFD (cm) | 8.00 (0.00-16.00) |  |  |  |
| PWD (cm) | 0.00 (0.00-6.00) |  |  |  |
| Chest expansion (cm) | 3.55 (2.00-5.00) |  |  |  |
| Schober test (cm) | 7.00 (4.00-9.50) |  |  |  |
| BASFI | 0.20 (0.00-1.00) |  |  |  |
| BASDAI | 1.71 (1.13-2.57) |  |  |  |
| ASDAS | 1.71 (1.27-2.42) |  |  |  |
| Disease duration (month) | 72.00 (12.00-156.00) |  |  |  |
| Treatment (n, %) |  |  |  |  |
| NSAIDs | 33 (82.5%) |  |  |  |
| TNF-α inhibitor | 24 (60.0%) |  |  |  |

Note: AS, ankylosing spondylitis; ASDAS, Ankylosing Spondylitis Disease Activity Score; BASDAI, Bath Ankylosing Spondylitis Disease Activity Index; BASFI, Bath Ankylosing Spondylitis Functional Index; CRP, C-reactive protein; ESR, erythrocyte sedimentation rate; FFD, Finger-floor distance; HC, healthy control; LYM, lymphocyte; MON, monocyte; NSAIDs, nonsteroidal anti-inflammatory drugs; PWD, pillow wall distance. Data were presented as mean ± SD or median (*P_25_, P_75_*).

Table S8 Correlation between Piezo1 expression levels and clinical indicators in patients with AS

| Indicators | *r_s_* | *P* value |
| --- | --- | --- |
| ESR | -0.062 | 0.716 |
| CRP | -0.133 | 0.432 |
| FFD | 0.178 | 0.277 |
| PWD | 0.323 | **0.041** |
| BASDAI | 0.259 | 0.106 |
| BASFI | 0.392 | **0.012** |
| ASDAS-ESR | -0.084 | 0.605 |
| Disease duration | 0.338 | **0.041** |

Note: *r_s_*: Spearman's correlation coefficient; *P* values with bold were considered statistically significant differences. AS, ankylosing spondylitis; ASDAS, Ankylosing Spondylitis Disease Activity Score; BASDAI, Bath Ankylosing Spondylitis Disease Activity Index; BASFI, Bath Ankylosing Spondylitis Functional Index; CRP, C-reactive protein; ESR, erythrocyte sedimentation rate; FFD, Finger-floor distance; PWD, pillow wall distance.

Table S9 Comparison of body weight among mouse groups at different time points.

| Weight | Group | | | *P*  (Con vs. Mod) | *P*  (Mod vs. GsM) |
| --- | --- | --- | --- | --- | --- |
|  | Control | Model | GsMTx4 |  |  |
| 0w | 23.42±1.41 | 22.91±0.94 | 23.32±1.26 | 0.379 | 0.444 |
| 1w | 23.91±1.94 | 22.93±0.98 | 23.00±1.02 | 0.197 | 0.890 |
| 2w | 23.29±1.58 | 23.26±1.00 | 23.62±0.91 | 0.958 | 0.426 |
| 3w | 24.04±1.99 | 23.51±0.74 | 23.96±0.79 | 0.463 | 0.237 |
| 4w | 24.47±1.71 | 23.93±0.87 | 23.56±0.86 | 0.417 | 0.368 |
| 5w | 25.06±1.77 | 23.89±0.80 | 24.33±1.00 | 0.091 | 0.313 |
| 6w | 24.67±1.95 | 24.07±0.93 | 25.31±1.14 | 0.417 | 0.022 |
| 7w | 25.29±2.03 | 24.11±1.03 | 24.08±1.02 | 0.140 | 0.946 |
| 8w | 25.78±2.33 | 24.42±1.06 | 25.20±1.17 | 0.131 | 0.159 |
| 9w | 25.80±1.96 | 24.82±1.09 | 25.53±1.06 | 0.210 | 0.181 |
| 10w | 26.47±1.90 | 25.47±1.49 | 26.44±1.09 | 0.233 | 0.133 |
| 11w | 27.04±2.37 | 25.73±1.70 | 26.40±1.76 | 0.197 | 0.426 |
| 12w | 27.11±2.01 | 25.96±1.76 | 26.91±1.22 | 0.213 | 0.200 |
| 13w | 27.47±2.59 | 26.09±1.55 | 26.98±2.21 | 0.189 | 0.338 |
| 14w | 28.07±2.61 | 26.76±1.86 | 27.64±2.06 | 0.238 | 0.350 |
| 15w | 28.13±2.69 | 26.67±1.85 | 27.16±1.34 | 0.197 | 0.531 |
| 16w | 28.56±2.71 | 26.91±1.77 | 28.09±1.56 | 0.147 | 0.154 |
| 17w | 29.29±2.91 | 26.82±1.65 | 27.33±1.85 | **0.042** | 0.545 |
| 18w | 28.89±2.67 | 26.84±2.28 | 28.36±1.52 | 0.100 | 0.117 |
| 19w | 28.76±2.87 | 26.71±1.94 | 28.09±1.67 | 0.096 | 0.126 |
| 20w | 29.30±2.66 | 26.87±2.07 | 28.31±1.56 | **0.046** | 0.114 |

Note: *P* values with bold were considered statistically significant differences.

Table S10 Comparison of peripheral arthritis scores among mouse groups at different time points.

| Peripheral Arthritis Score | Group | | | *P*  (Con vs. Mod) | *P*  (Mod vs. GsM) |
| --- | --- | --- | --- | --- | --- |
|  | Control | Model | GsMTx4 |  |  |
| 0w | 0.00±0.00 | 0.00±0.00 | 0.00±0.00 | 1.000 | 1.000 |
| 1w | 0.00±0.00 | 0.00±0.00 | 0.00±0.00 | 1.000 | 1.000 |
| 2w | 0.00±0.00 | 0.00±0.00 | 0.00±0.00 | 1.000 | 1.000 |
| 3w | 0.00±0.00 | 0.67±1.12 | 0.56±0.88 | 0.093 | 0.818 |
| 4w | 0.00±0.00 | 0.44±0.73 | 0.67±1.00 | 0.085 | 0.597 |
| 5w | 0.00±0.00 | 0.67±0.87 | 0.44±0.88 | **0.035** | 0.597 |
| 6w | 0.00±0.00 | 1.33±0.50 | 1.22±0.44 | **<0.001** | 0.624 |
| 7w | 0.00±0.00 | 2.56±1.13 | 3.00±1.00 | **<0.001** | 0.390 |
| 8w | 0.00±0.00 | 3.78±0.97 | 3.89±1.05 | **<0.001** | 0.819 |
| 9w | 0.00±0.00 | 6.22±2.11 | 6.89±1.45 | **<0.001** | 0.446 |
| 10w | 0.00±0.00 | 8.00±0.71 | 8.44±1.94 | **<0.001** | 0.528 |
| 11w | 0.22±0.44 | 9.11±1.45 | 9.44±1.67 | **<0.001** | 0.657 |
| 12w | 0.22±0.44 | 10.78±2.91 | 10.11±1.17 | **<0.001** | 0.532 |
| 13w | 0.44±0.53 | 12.67±2.18 | 10.67±2.00 | **<0.001** | 0.060 |
| 14w | 0.56±0.53 | 12.89±1.76 | 10.00±1.66 | **<0.001** | **0.003** |
| 15w | 0.56±0.53 | 13.33±2.00 | 10.11±1.61 | **<0.001** | **0.002** |
| 16w | 0.56±0.53 | 13.11±1.69 | 9.78±2.11 | **<0.001** | **0.002** |
| 17w | 0.56±0.53 | 12.56±2.40 | 8.78±1.92 | **<0.001** | **0.002** |
| 18w | 0.78±0.83 | 11.89±1.27 | 9.22±1.56 | **<0.001** | **0.001** |
| 19w | 0.78±0.83 | 11.44±2.07 | 8.22±1.56 | **<0.001** | **0.002** |
| 20w | 0.78±0.83 | 11.11±1.76 | 7.22±1.39 | **<0.001** | **<0.001** |

Note: *P* values with bold were considered statistically significant differences.

Table S11 Sequence list of gene primers

| Gene | Forward，5'to3' | Reverse，5'to3' |
| --- | --- | --- |
| Piezo1 | CAATGAGGAGGCCGACTACC | GCACTCCTGCAGTTCGATGA |
| MDFIC | GGAAATCCTTCGGATGGTGAACTC | CAAGCAAGCCAGGATACAGTGGACAC |
| ATG3 | GATGGCGGATGGGTAGATACA | TCTTCACATAGTGCTGAGCAATC |
| ATG5 | CACAAGCAACTCTGGATGGGATTG | GCAGCCACAGGACGAAACAG |
| ATG12 | TAGAGCGAACACGAACCATCC | CACTGCCAAAACACTCATAGAGA |
| Beclin 1 | CGACAACGGCTCCGGCATGT | TGCCGTGCTCGATGGGGTACT |
| SQSTM1/P62 | GCACCCCAATGTGATCTGC | CGCTACACAAGTCGTAGTCTGG |
| LC3B | GAGAAGCAGCTTCCTGTTCTGG | GTGTCCGTTCACCAACAGGAAG |
| CD80 | AAACTCGCATCTACTGGCAAA | GGTTCTTGTACTCGGGCCATA |
| IL-6 | AACCTGAACCTTCCAAAGATGG | TCTGGCTTGTTCCTCACTACT |
| CD163 | TGAGCAGCACATGGGAGATTG | ACGATGAATTGCACGAGGACA |
| CD209 | AGTGACTCCAAGGAACCAAGAC | GATCGCGTCTTGCCTGGATT |
| ALP | ACCACCACGAGAGTGAACCA | CGTTGTCTGAGTACCAGTCCC |
| RUNX2 | TGGTTACTGTCATGGCGGGTA | TCTCAGATCGTTGAACCTTGCTA |
| BMP2 | TTCGGCCTGAAACAGAGACC | CCTGAGTGCCTGCGATACAG |
| OCN | CACTCCTCGCCCTATTGGC | CCCTCCTGCTTGGACACAAAG |
| IL-1β | TGAAATGATGGCTTATTACAGTGG | GTAGTGGTGGTCGGAGATTCGTAG |
| GAPDH | GTCTCCTCTGACTTCAACAGCG | ACCACCCTGTTGCTGTAGCCAA |

Table S12 Sequences of si-Piezo1.

| Gene | Sense (5′-3′) | Antisense (5′-3′) |
| --- | --- | --- |
| si-Piezo1-P1 | CCAAGUACUGGAUCUAUGU | ACAUAGAUCCAGUACUUGG |
| si- Piezo1-P2 | CUCAAGUACUUCAUCAACU | AGUUGAUGAAGUACUUGAG |
| si- Piezo1-P3 | CCAAGAAGUACAAUCAUCU | AGAUGAUUGUACUUCUUGG |
| si-control | UUCUCCGAACGUGUCACGU | ACGUGACACGUUCGGAGAA |


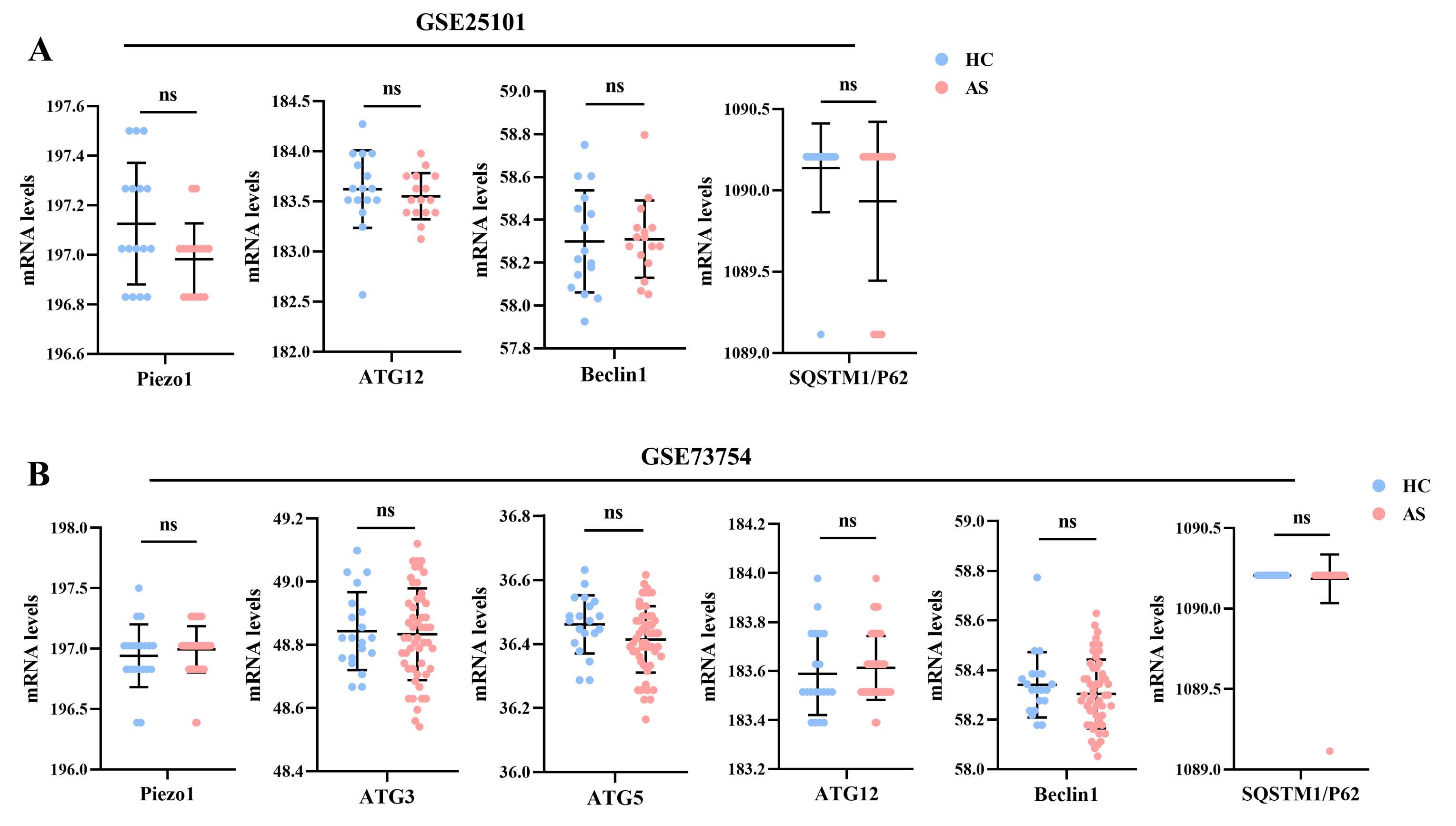


Figure S1 Levels of Piezo1 and autophagy-related genes in AS patients. (A) Relative expression of piezo1 and autophagy-related genes in the GSE25101 (HC, n = 16; AS, n = 16) whole-blood genome sequencing dataset. (B) Relative expression of piezo1 and autophagy-related genes in the GSE73754 (HC, n = 20; AS, n = 52) whole-blood genome sequencing dataset. NS, not significant. AS, ankylosing spondylitis; HC, healthy control.


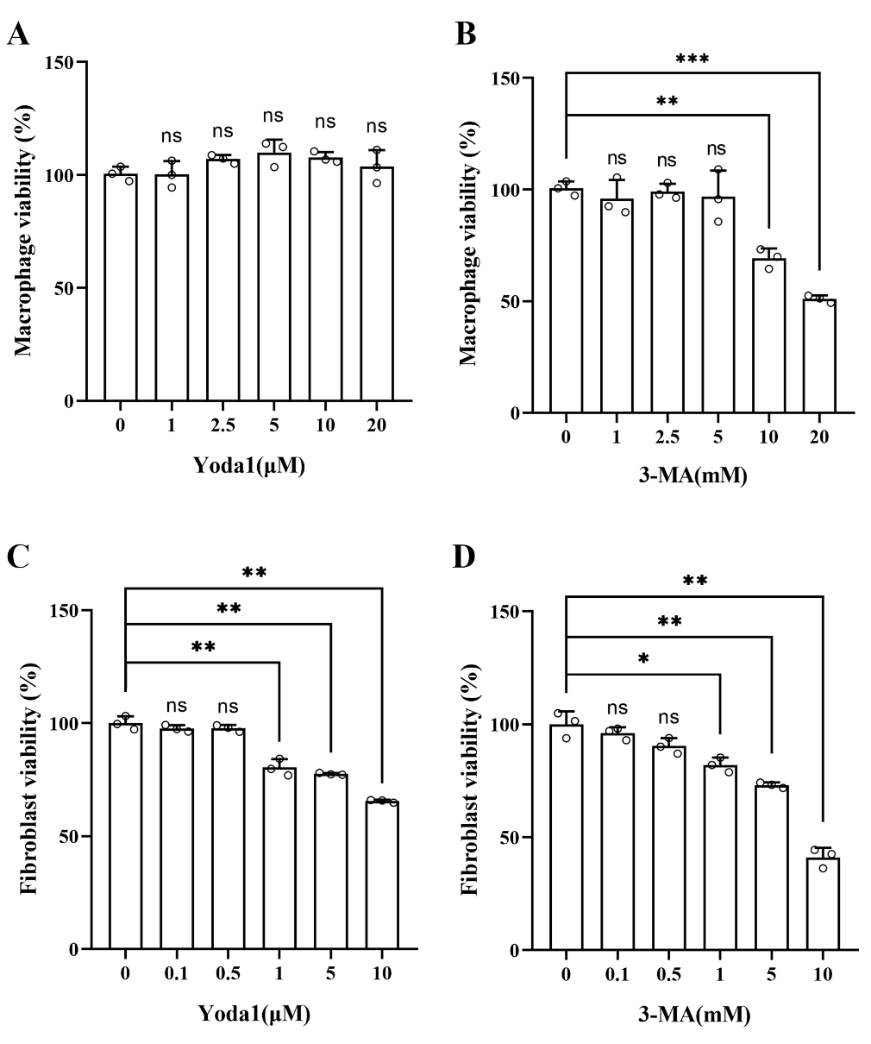


Figure S2 Cell viability of monocyte-macrophages (A, B) and FLS (C, D) following treatment with varying concentrations of Yoda1 or 3-MA, as assessed using the CCK-8 assay. n=3. Data shown as mean±SD. **P* < 0.05, ***P* < 0.01, ****P* < 0.001, NS, not significant.


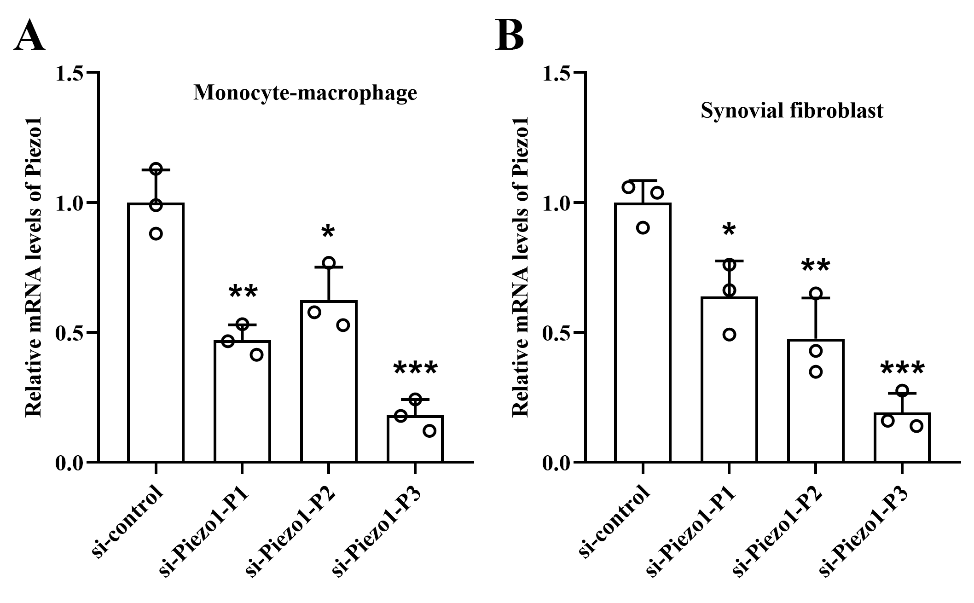


Figure S3. Relative mRNA expression levels of Piezo1 in monocyte-derived macrophages (A) and FLS (B) following transfection with si-Piezo1. Data are presented as mean ± SD from three independent experiments. **P* < 0.05, ***P* < 0.01, ****P* < 0.001 vs. si-control.

**Supplementary Methods**

**CCK-8 assay**

After digestion with an appropriate amount of trypsin, monocyte-macrophages and FLS were adjusted to 2 × 10⁴ cells/mL and seeded into 96-well plates at 100 μL per well, with three replicates per group. Once the cells adhered, the medium was replaced with Yoda1 or 3-MA. After the designated intervention period, 10 μL of CCK8 reagent was added per well, and absorbance at 450 nm was measured after 3 hours using a microplate reader (Figure S1).

**RT-qPCR**

RT-qPCR was conducted with the PrimeScript™ RT kit (Takara) on a LightCycler 480 II PCR instrument. Relative expression levels were calculated using the 2^-ΔΔCt^ method and normalized. Primer sequences are provided in Table S11.

**Immunohistochemistry (IHC) staining**

After fixation and decalcification of synovial and spinal tissues, the samples were dehydrated through a gradient of ethanol, immersed in xylene, embedded in paraffin, and sectioned into 4 µm slices. The tissue sections were then baked, dewaxed, and rehydrated using a gradient ethanol series. For antigen repairing, sections were incubated in EDTA buffer for 15 minutes, washed three times with PBS, and treated with 3% hydrogen peroxide for 25 minutes. Following another PBS wash, sections were blocked with 3% BSA for 30 minutes. The sections were then incubated overnight at 4℃ with primary antibodies: Piezo1 (Proteintech, 15939-1-AP, 1:100), LC3B (Proteintech, 18725-1-AP, 1:200), and SQSTM1/P62 (Proteintech, 18420-1-AP, 1:500). After three PBS washes, the sections were incubated with HRP-labeled secondary antibody for 1 hour at room temperature, stained with hematoxylin following DAB development, and dehydrated through a gradient ethanol series and mounted with neutral gum, and the staining results were observed and recorded under a light microscope.

**Immunofluorescence (IF) staining**

Tissue sections were incubated overnight at 4℃ with primary antibodies against the following antigens: Piezo1 (15939-1-AP, 1:100), LC3B (18725-1-AP, 1:200), CD68 (25747-1-AP, 1:100), F4/80 (28463-1-AP, 1:100), Vimentin (10366-1-AP, 1:200), TNF-α (17590-1-AP, 1:100), and IL-6 (21865-1-AP, 1:100) (Proteintech). Corresponding Alexa Fluor–conjugated secondary antibodies were used for visualization. Nuclei were counterstained with DAPI. Staining was examined with a fluorescence microscope, and positive cells were quantified using ImageJ software.

**GEO expression profile analysis**

Quantile normalization of four primary datasets (GSE25101, GSE73754, GSE39340, and GSE41038) was performed using R (version 4.2.1). Integration of the synovial tissue datasets GSE39340 and GSE41038 included batch effect correction using the "sva" package and data preprocessing with the "limma" package, which encompassed background correction, normalization, and log transformation. Principal component analysis (PCA) was used to evaluate batch effect removal, with PCA plots shown before and after correction. Notably, GSE39340 included five AS samples (with two outliers excluded from analysis), while GSE41038 comprised two AS samples and four controls. All GEO data processing and visualization were conducted using R (version 4.2.1).

**Determination of [Ca^2+^]**

To measure intracellular Ca²⁺ concentrations, monocyte-macrophage cells were incubated with Fluo-4 AM (Beyotime) for 30 minutes at 37℃, protected from light. Fluorescence microscopy was used to capture live-cell images.

**Western blotting**

Cells or tissue homogenates were lysed using an appropriate amount of lysis buffer and crushed by ultrasonic waves, and centrifuged at 14,000×g for 10 min at 4℃. Protein concentration was determined using a BCA protein quantification kit (Thermo). Proteins were separated by SDS-PAGE and transferred to PVDF membranes. Membranes were incubated overnight at 4℃ with the following primary antibodies: Piezo1 (Catalog: 15939-1-AP, 1:1000, Proteintech), LC3B (Catalog: 18725-1-AP, 1:1000, Proteintech), SQSTM1/P62 (Catalog: 18420-1-AP, 1:5000, Proteintech), ATG3 (Catalog: 10494-1-AP, 1:10000, Proteintech), IL-6 (Catalog: 21865-1-AP, 1:1000, Proteintech), GAPDH (Catalog: 21865-1-AP, 1:1000, Proteintech), and β-Actin (Catalog: YT0099, 1:5000, ImmunoWay). Membranes were incubated with the secondary antibody for 1 hour at room temperature. Protein bands were visualized using a chemiluminescent gel imaging system. All WB experiments were performed at least three times.

**ELISA**

ELISA kits (R&D Systems) were used to quantify TNF-α, IL-6, IL-8, IL-17A, and IL-23 in cell supernatants and mouse serum, following the manufacturer's instructions. Optical density (OD) was measured at 450 nm and corrected by subtracting the 570 nm OD value. The concentrations of inflammatory factors were then calculated based on a standard curve.

**ALP staining**

ALP staining was conducted using the BCIP/NBT ALP Chromogenic Kit (Beyotime) per the manufacturer’s protocol. Cells were incubated with the staining solution for 30 min at 37°C in the dark, and results were observed under a light microscope.

**H&E and SOFG staining**

Tissue sections were dewaxed and hydrated with gradient alcohol, and stained with hematoxylin and 1% eosin or impregnated with Safranin O and fast green. Following staining, the sections were dehydrated, cleared, and mounted with neutral gum. The staining results were observed and recorded using a light microscope.

**Micro-CT**

After euthanasia, mouse spines were harvested and fixed in 4% paraformaldehyde. Each spine was placed in a sample scanning box and imaged using a high-resolution Micro-CT system (NEMO, NMC-100) with the following parameters: tube voltage of 90 kV, tube current of 0.09 mA, scanning resolution of 10 μm, and CT queue value of 1040. Following scanning, three-dimensional reconstruction was performed using the onboard Recon software. Bone-related indices and lumbar vertebral spacing in 2D sagittal planes were analyzed using Avatar software.
