# Supplementary figures and images for: Piezo1-mediated autophagy promotes immune-inflammatory responses in ankylosing spondylitis
Source: Cell Death Dis. 2026 Jan 8;17(1):12. doi: 10.1038/s41419-025-08230-7 (PMC12783605; doi:10.1038/s41419-025-08230-7)

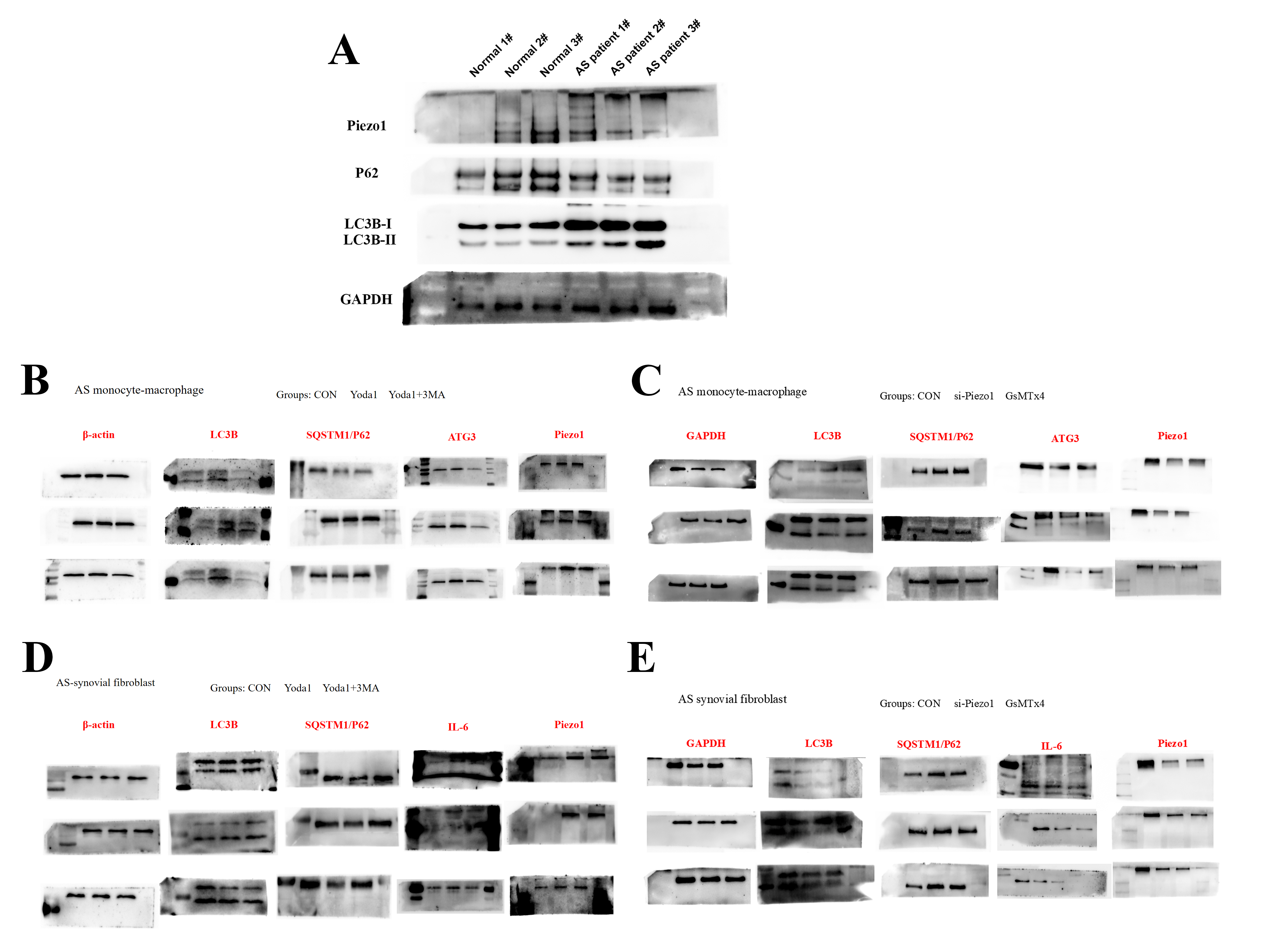

Supplement: Supplementary file 2 — Original western blots [file 41419_2025_8230_MOESM2_ESM.tif]
